# Supplementary material for: Fine-Tuning Unifies Foundational Machine-Learned Interatomic Potential Architectures at ab initio Accuracy
Source: J Phys Chem Lett. 2026 Mar 4;17(11):3152–62. doi: 10.1021/acs.jpclett.5c03801 (PMC13007020; doi:10.1021/acs.jpclett.5c03801)
Supplement: Supplementary file 1 [file jz5c03801_si_001.pdf]

Supporting Information: Fine-Tuning Unifies Foundational Machine-learned Interatomic Potential Architectures at ab initio Accuracy

Jonas Hänseroth,* Aaron Flötotto, Muhammad Nawaz Qaisrani, and Christian Dreßler

Theoretical Solid State Physics, Institute of Physics, Technische Universität Ilmenau, 98693 Ilmenau, Germany

E-mail: jonas.haenseroth@tu-ilmenau.de

Table S1: Absolute force and energy errors for foundation and fine-tuned models across all evaluated systems. Errors: force (eV Å^{-1}), energy per atom (eV).

System	Model	MACE-MP-0		GRACE-1L-OAM		SevenNet-0		MatterSim-Large		ORB-v2	
		Force	Energy	Force	Energy	Force	Energy	Force	Energy	Force	Energy
CsH_2PO_4	Foundation	0.2411	307.68	0.2782	307.69	0.2031	307.69	0.1960	307.69	0.1916	307.69
	Fine-tuned	0.0543	0.00041	0.0631	0.00017	0.0316	0.00096	0.032	0.00050	0.0378	0.00192
$Cs_7(H_4PO_4)(H_2PO_4)_8$	Foundation	0.2310	292.84	0.2787	292.85	0.2039	292.84	0.2169	292.85	0.1966	292.85
	Fine-tuned	0.0364	0.00015	0.0699	0.00017	0.0414	0.00077	0.0363	0.00237	0.0480	0.00249
L-pyroglutamate-NH_4	Foundation	0.4484	139.42	0.4370	139.43	0.3584	139.44	0.3476	139.44	0.3295	139.43
	Fine-tuned	0.0333	0.00153	0.0403	0.00013	0.0211	0.00183	0.0232	0.00320	0.0404	0.00075
PhOH in H_2O	Foundation	0.1562	149.64	0.1750	149.64	0.1551	149.64	0.1607	149.64	0.1528	149.63
	Fine-tuned	0.0261	0.00052	0.0485	0.00022	0.0388	0.00312	0.0490	0.00940	0.0383	0.00150
KOH in H_2O	Foundation	0.0999	161.61	0.1294	161.62	0.0851	161.62	0.0954	161.62	0.0910	161.61
	Fine-tuned	0.0351	0.00341	0.0485	0.00213	0.0193	0.00216	0.0354	0.00594	0.0426	0.00272
$Li_{13}Si_4$	Foundation	0.1333	177.21	0.0923	177.23	0.1660	177.22	0.1063	177.23	0.0861	177.22
	Fine-tuned	0.0220	0.00310	0.0190	0.00143	0.0151	0.00186	0.0313	0.00234	0.0327	0.00405
MoS_2	Foundation	0.5103	807.51	0.2587	807.52	0.5448	807.51	0.2317	807.53	0.4510	807.55
	Fine-tuned	0.0299	0.00109	0.02299	0.00350	0.0284	0.00013	0.0175	0.00023	0.0431	0.00136

Table S2: Computing time in minutes for fine-tuning across different MLIP frameworks and chemical systems per 100 epochs on one NVIDIA A100.

System	MACE	GRACE	SevenNet	MatterSim	ORB
CDP	134.0	40.9	373.0	342.1	77.7
CPP	167.0	36.7	364.7	456.0	108.0
L-PyroNH_4	37.5	12.2	188.5	128.0	44.0
PhOH	42.3	16.8	45.5	45.4	8.8
KOH	85.0	26.1	359.2	338.0	150.5
$Li_{13}Si_4$	67.0	21.0	164.0	178.6	58.7

Table S3: Computing time for 10,000 molecular dynamics steps and fine-tuning 100 epochs (2,000 data points) of a system containing 512 atoms on one NVIDIA A100.

Task	MACE	MACE+cueq	GRACE
MD foundation (s)	412.6	385.1	292.2
MD fine-tuned (s)	390.2	383.5	312.6
Fine-tuning model (min)	134.0	51.8	40.9

Task	SevenNet	MatterSim	ORB
MD foundation (s)	549.0	915.6	131.6
MD fine-tuned (s)	555.0	904.8	131.6
Fine-tuning model (min)	373.0	342.1	77.7

Table S4: Hyperparameters used for fine-tuning across different MLIP frameworks and chemical systems. Learning rates, force weights, and epoch counts show both framework-specific preferences and system-dependent requirements.

System	Framework	Learning Rate	Force Weight	Batch Size	Epochs
CsH_2PO_4	MACE	0.01	100	5	200
	GRACE	0.002	150	4	2000
	SevenNet	0.01	1	5	250
	MatterSim	0.0005	0.5	5	500
	ORB	0.0003	0.5	4	1650
$Cs_7(H_4PO_4)(H_2PO_4)_8$	MACE	0.01	100	5	200
	GRACE	0.002	50	4	2500
	SevenNet	0.004	1	4	300
	MatterSim	0.0005	0.5	5	500
	ORB	0.0003	1	4	400
L-pyroglutamate-NH_4	MACE	0.01	10	5	200
	GRACE	0.002	100	4	1000
	SevenNet	0.01	100	4	200
	MatterSim	0.0005	0.5	5	500
	ORB	0.0003	0.5	4	400
PhOH in H_2O	MACE	0.01	10	5	200
	GRACE	0.002	100	4	500
	SevenNet	0.004	100	4	400
	MatterSim	0.0005	0.25	5	500
	ORB	0.0002	0.25	8	800
KOH in H_2O	MACE	0.01	100	5	200
	GRACE	0.001	5	4	500
	SevenNet	0.01	100	4	200
	MatterSim	0.0005	0.5	5	500
	ORB	0.0003	1	4	200
$Li_{13}Si_4$	MACE	0.01	10	5	200
	GRACE	0.002	100	4	500
	SevenNet	0.004	50	4	200
	MatterSim	0.0005	0.5	5	350
	ORB	0.0003	0.75	4	1250
MoS_2	MACE	0.01	100	5	200
	GRACE	0.001	100	4	1000
	SevenNet	0.01	1	5	400
	MatterSim	0.001	10	5	500
	ORB	0.0003	0.5	4	750

System A: CsH_2PO_4

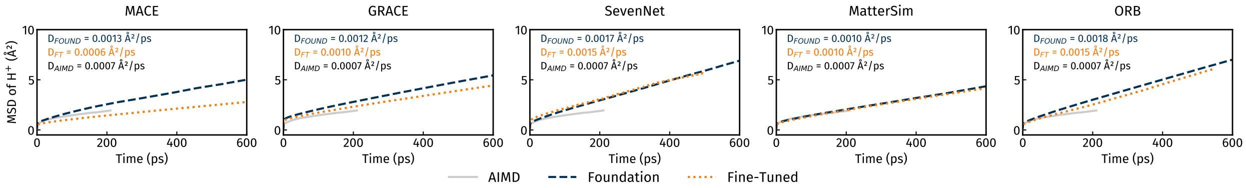

Figure S1: Mean-squared displacements of H^+ in CsH_2PO_4 computed using different MLIP frameworks. Results from the foundation model and the fine-tuned foundation model are compared against AIMD reference data.

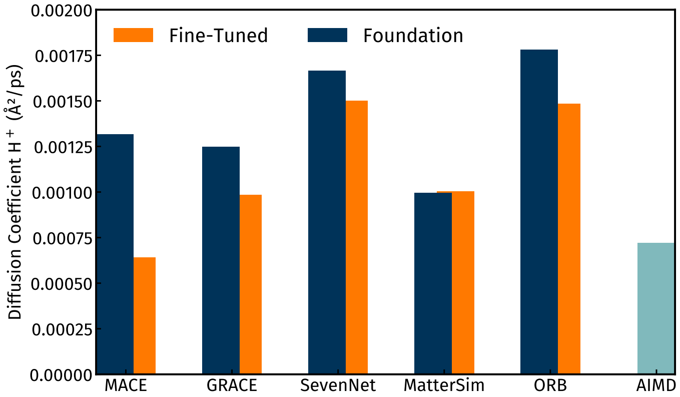

Figure S2: Diffusion coefficients of H^+ in CsH_2PO_4 computed using different MLIP frameworks from the mean-square displacements (see Figure S1). Results from the foundation model and the fine-tuned foundation model are compared against AIMD reference data.

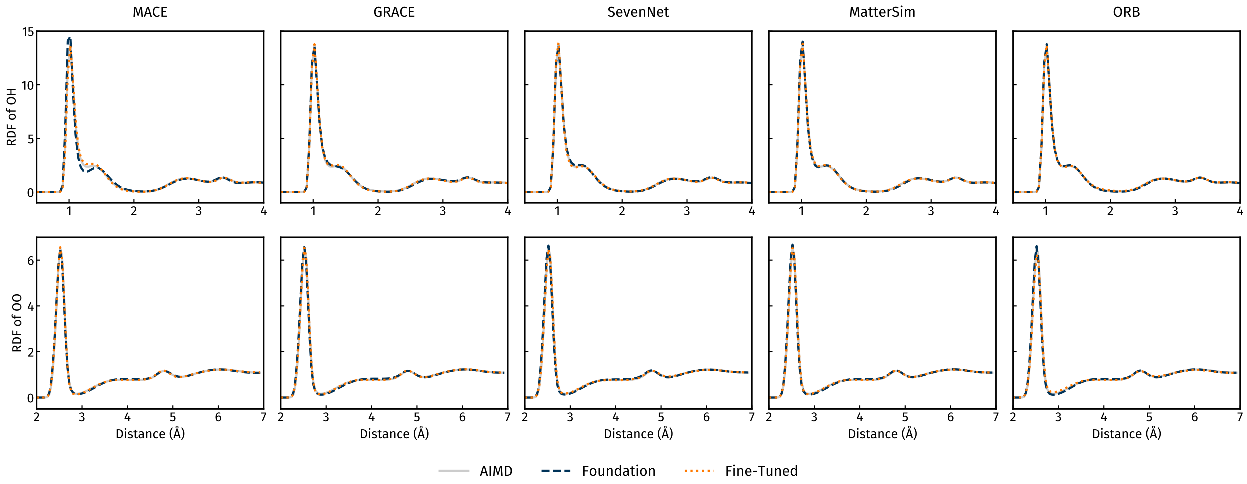

Figure S3: Radial-distribution functions of O-H and O-O in CsH_2PO_4 computed using different MLIP frameworks. Results from the foundation model and the fine-tuned foundation model are compared against AIMD reference data.

System B: $Cs_7(H_4PO_4)(H_2PO_4)_8$

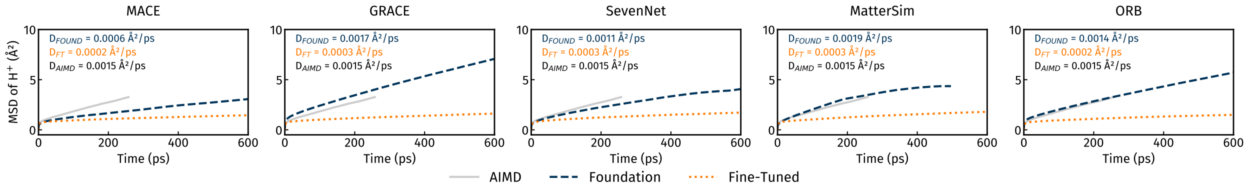

Figure S4: Mean-squared displacements of H^+ in $Cs_7(H_4PO_4)(H_2PO_4)_8$ computed using different MLIP frameworks. Results from the foundation model and the fine-tuned foundation model are compared against AIMD reference data.

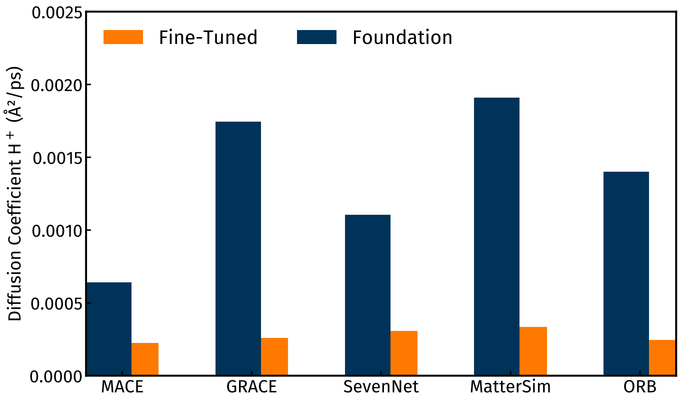

Figure S5: Diffusion coefficients of H^+ in $Cs_7(H_4PO_4)(H_2PO_4)_8$ computed using different MLIP frameworks from the mean-square displacements (see Figure S4). Results are shown for the foundation model and the fine-tuned foundation model. Reference AIMD data are not available, as AIMD simulations cannot provide converged diffusion coefficients for this system. For comparison, a recent MLIP study reported a diffusion coefficient of 0.0004 $Å^2$/ps at 510 K.[S1]

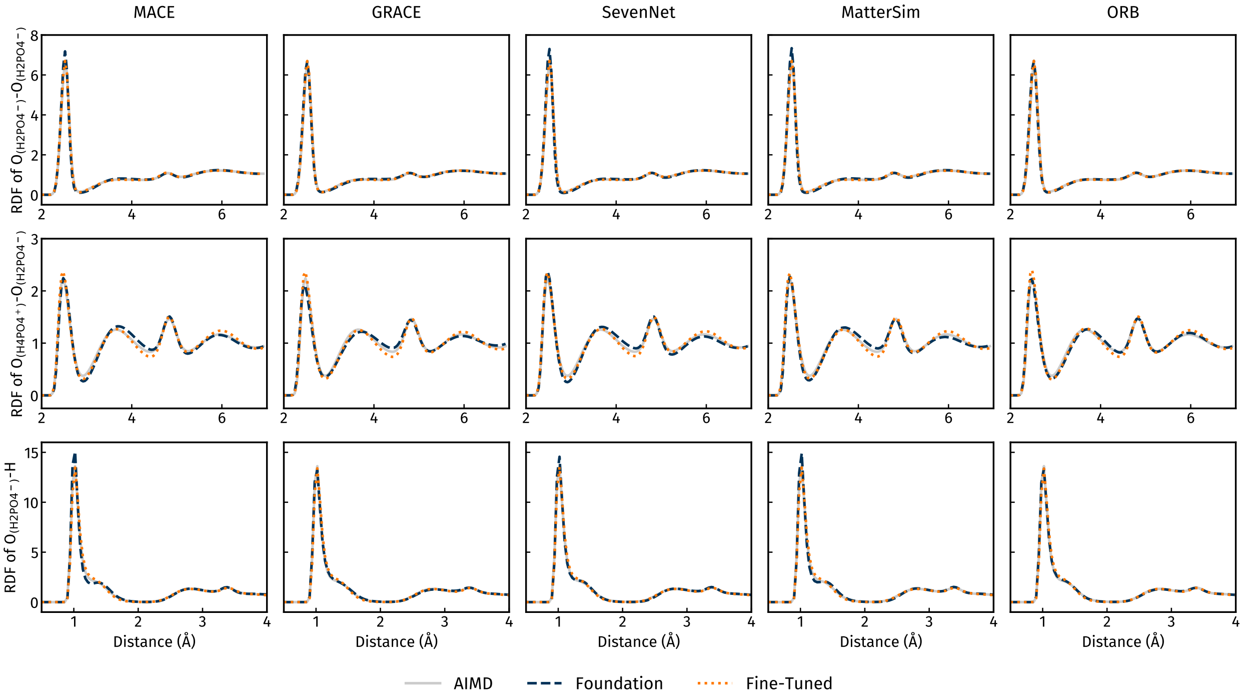

Figure S6: Radial-distribution functions of $O_{H_2PO_4}$-$O_{H_2PO_4}$-, $O_{H_4PO_4}$+-$O_{H_2PO_4}$- and $O_{H_2PO_4}$-H in $Cs_7(H_4PO_4)(H_2PO_4)_8$ computed using different MLIP frameworks. Results from the foundation model and the fine-tuned foundation model are compared against AIMD reference data.

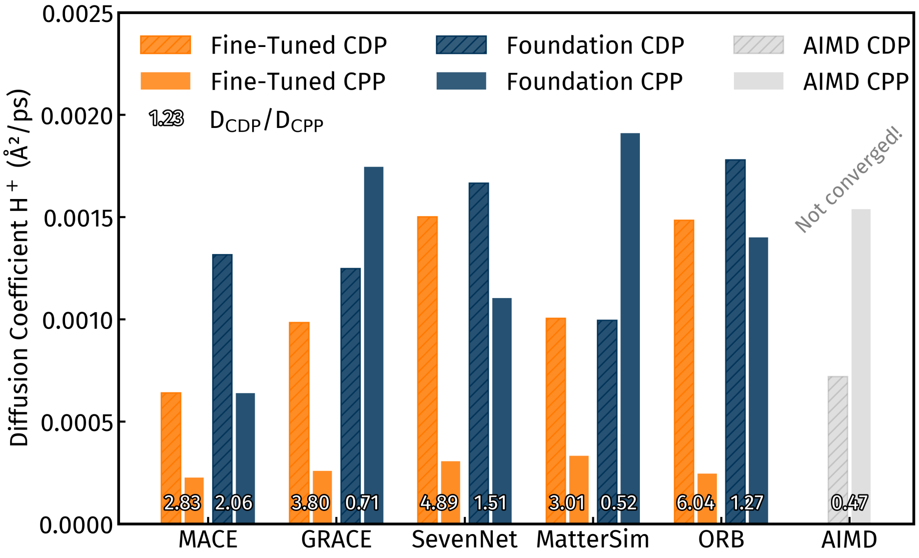

Figure S7: Diffusion coefficients comparison of H^+ in CsH_2PO_4 and $Cs_7(H_4PO_4)(H_2PO_4)_8$ computed using different MLIP frameworks from the mean-square displacements (see Figure S2 and Figure S5). Results are shown for the foundation model and the fine-tuned foundation model. The AIMD data result in non-converged diffusion coefficients. For comparison, a recent MLIP study reported a diffusion coefficient ratio of 4.[S1]

System C: $Li_{13}Si_4$

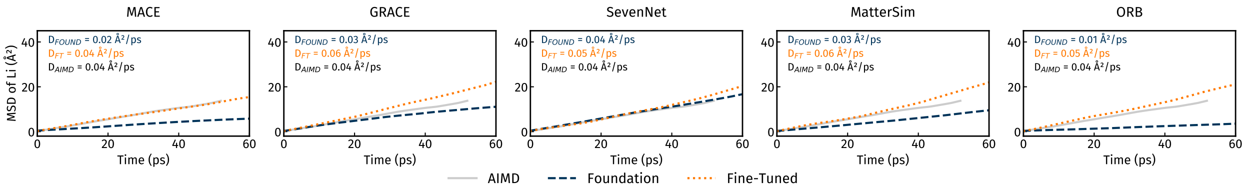

Figure S8: Mean-squared displacements of Li^+ in $Li_{13}Si_4$ computed using different MLIP frameworks. Results from the foundation model and the fine-tuned foundation model are compared against AIMD reference data.

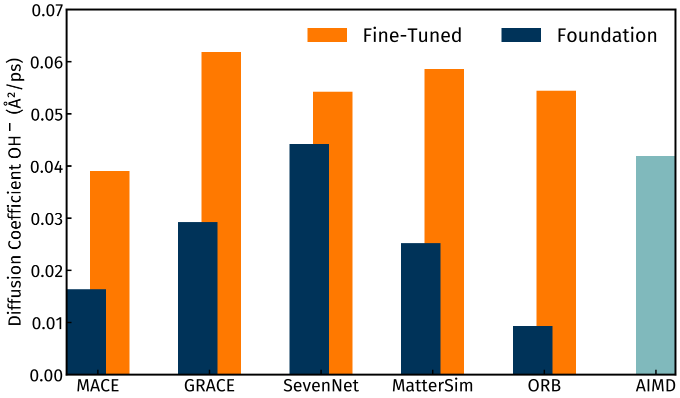

Figure S9: Diffusion coefficients of Li^+ in $Li_{13}Si_4$ computed using different MLIP frameworks from the mean-square displacements (see Figure S8). Results from the foundation model and the fine-tuned foundation model are compared against AIMD reference data.

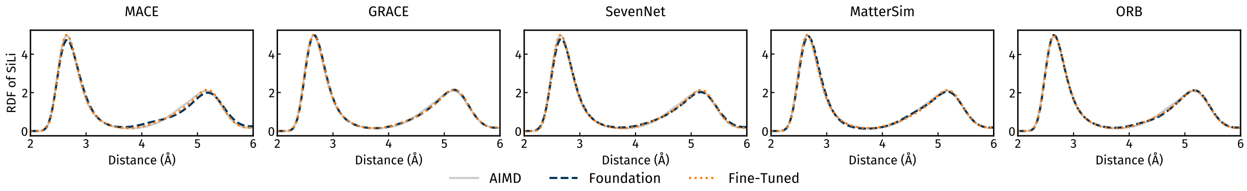

Figure S10: Radial-distribution functions of Si-Li in $Li_{13}Si_4$ computed using different MLIP frameworks. Results from the foundation model and the fine-tuned foundation model are compared against AIMD reference data.

System D: PhOH in H_2O

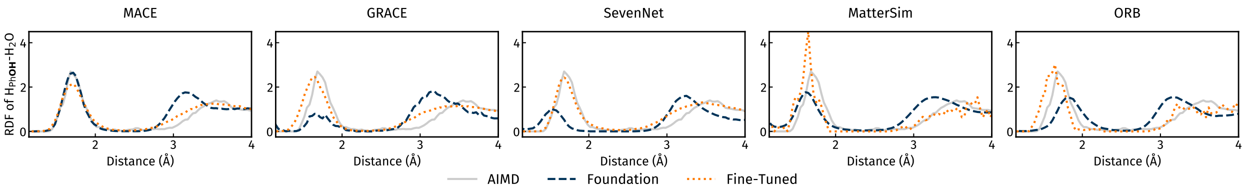

Figure S11: Radial-distribution functions of $H_{\text{Hydroxyl-Group}}$-$O_{\text{Water}}$ in PhOH in water computed using different MLIP frameworks. Results from the foundation model and the fine-tuned foundation model are compared against AIMD reference data.

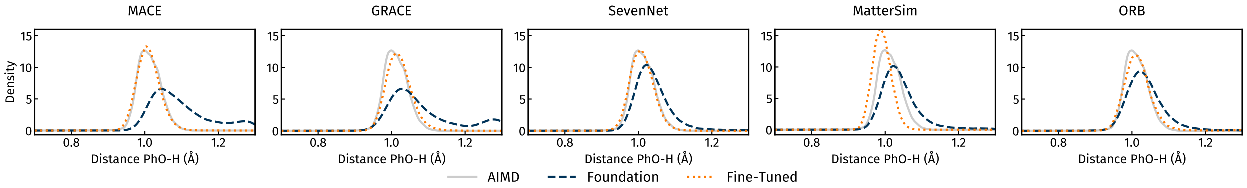

Figure S12: Distribution of the hydroxyl group O–H bond length in the phenol in water computed using different MLIP frameworks. Results from the foundation model and the fine-tuned foundation model are compared against AIMD reference data.

System E: KOH in H_2O

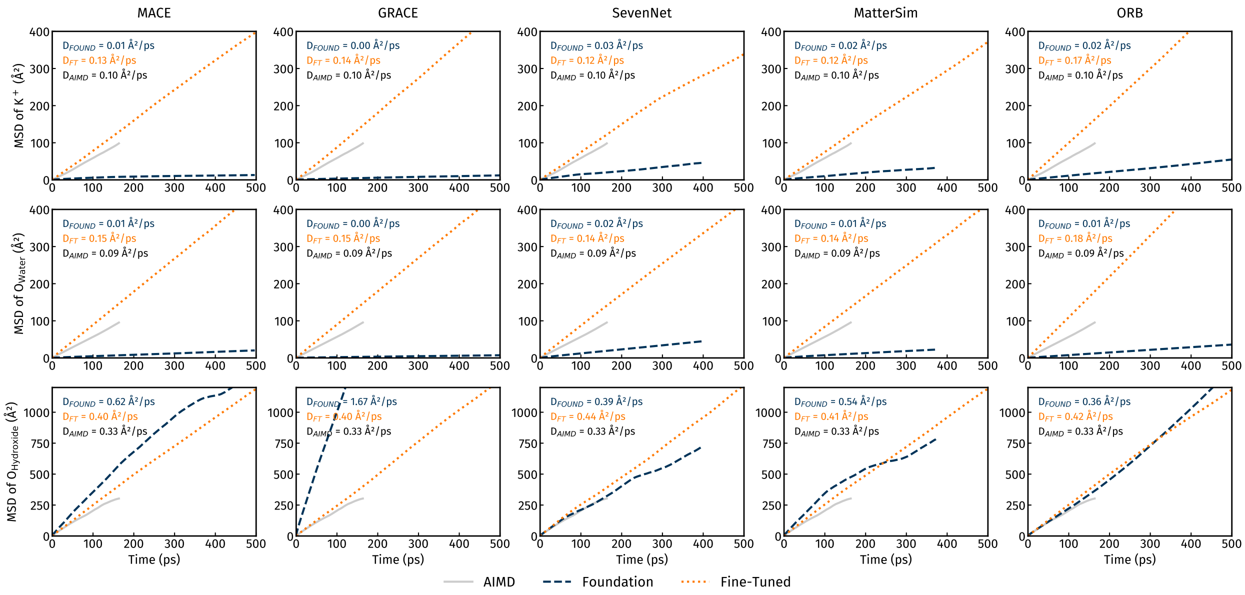

Figure S13: Mean-squared displacements of K^+, H_2O and OH^- in aqueous potassium hydroxide solution computed using different MLIP frameworks. Results from the foundation model and the fine-tuned foundation model are compared against AIMD reference data.

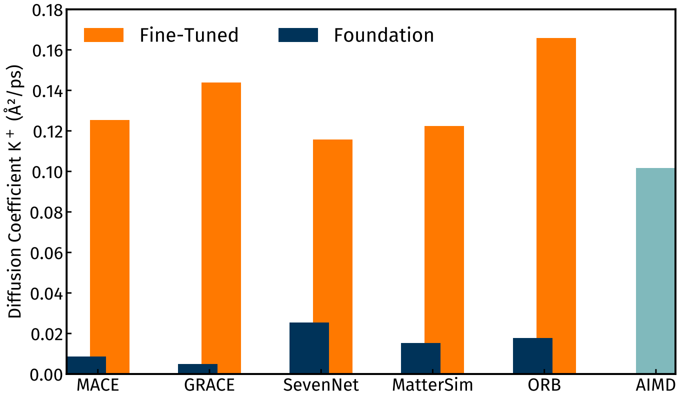

Figure S14: Diffusion coefficients of K^+ in aqueous potassium hydroxide solution computed using different MLIP frameworks from the mean-square displacements (see Figure S13). Results from the foundation model and the fine-tuned foundation model are compared against AIMD reference data.

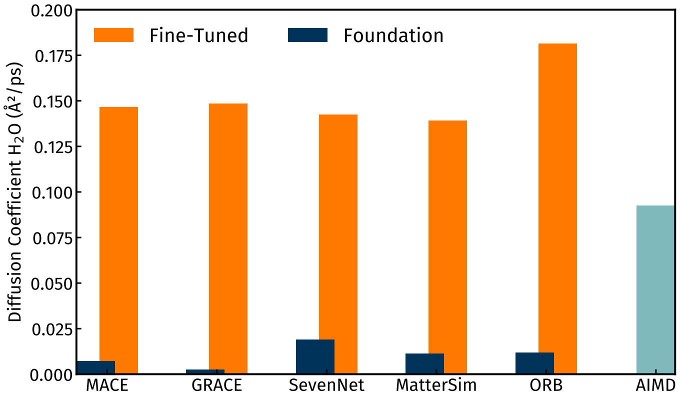

Figure S15: Diffusion coefficients of H_2O in aqueous potassium hydroxide solution computed using different MLIP frameworks from the mean-square displacements (see Figure S13). Results from the foundation model and the fine-tuned foundation model are compared against AIMD reference data.

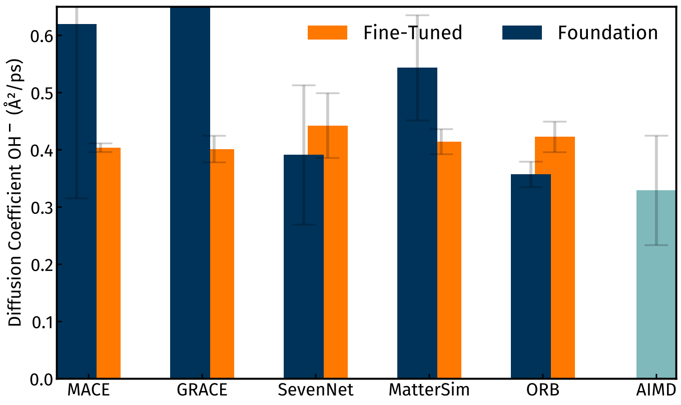

Figure S16: Diffusion coefficients of OH^- in aqueous potassium hydroxide solution computed using different MLIP frameworks from the mean-square displacements (see Figure S13). Results from the foundation model and the fine-tuned foundation model are compared against AIMD reference data.

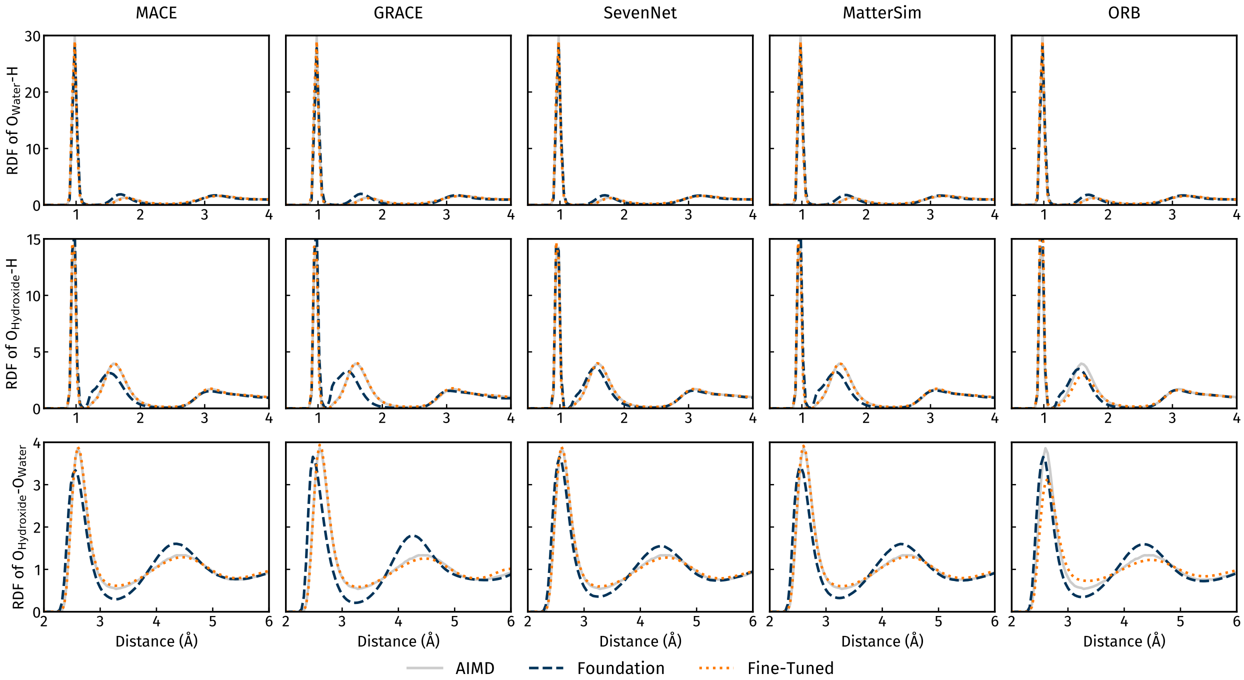

Figure S17: Radial-distribution functions of O_{Water}-H, $O_{Hydroxide}$-H and $O_{Hydroxide}$-O_{Water} in aqueous potassium hydroxide solution computed using different MLIP frameworks. Results from the foundation model and the fine-tuned foundation model are compared against AIMD reference data.

System E: L-pyroglutamate-NH_4

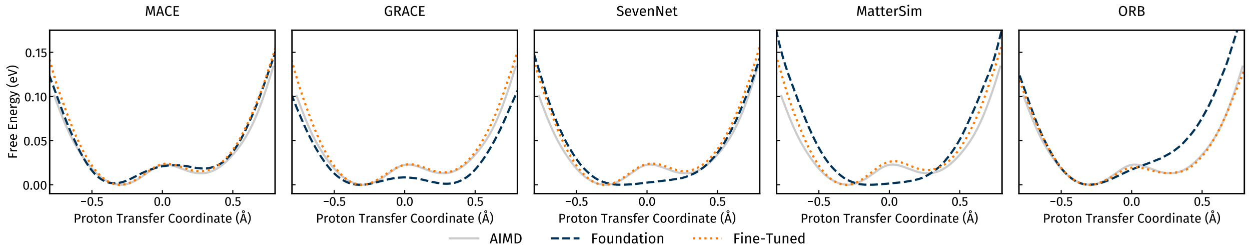

Figure S18: Free energy profiles along the proton transfer coordinate of the short-hydrogen-bond in L-pyroglutamate-NH_4 computed using different MLIP frameworks. Results from the foundation model and the fine-tuned foundation model are compared against AIMD reference data.

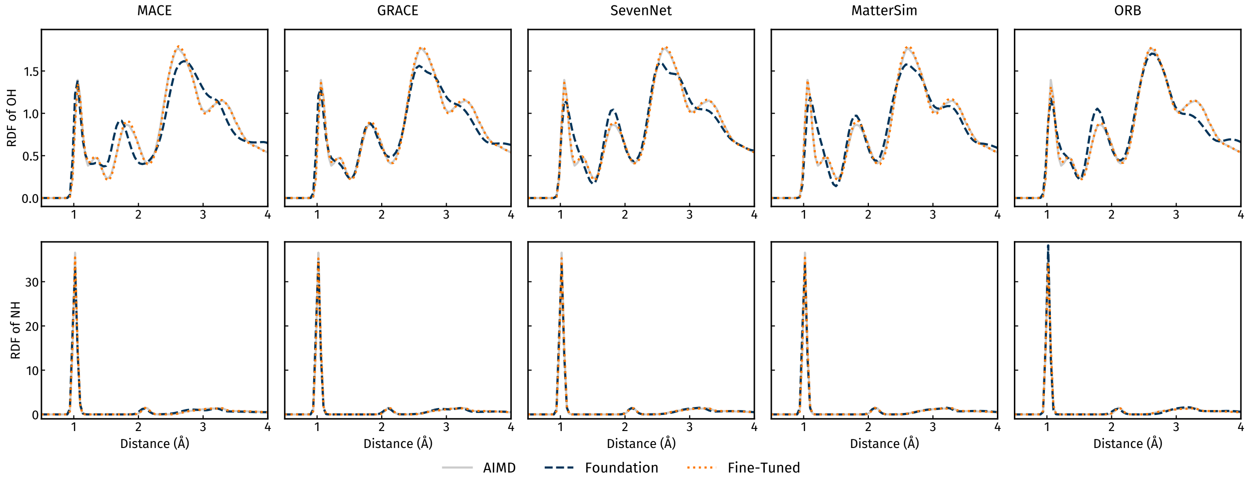

Figure S19: Radial-distribution functions of O-H and N-H in L-pyroglutamate-NH_4 computed using different MLIP frameworks. Results from the foundation model and the fine-tuned foundation model are compared against AIMD reference data.

System E: MoS_2

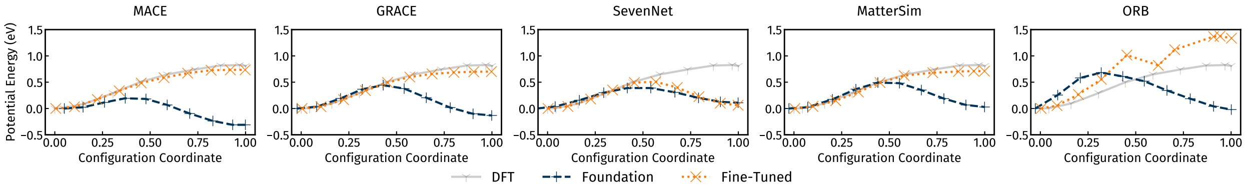

Figure S20: Potential energy curves for a sulfur jump into a sulfur vacancy cluster in MoS_2 computed using different MLIP frameworks. Results from the foundation model and the fine-tuned foundation model are compared against DFT reference data. Note: Fine-tuning attempts for the SevenNet foundation model did not yield models capable of reproducing the reference potential energy curve, even after hyperparameter optimization.

References

(S1) Grunert, M.; Großmann, M.; Hänseroth, J.; Flötotto, A.; Oumard, J.; Wolf, J. L.; Runge, E.; Dreßler, C. Modeling Complex Proton Transport Phenomena - Exploring the Limits of Fine-Tuning and Transferability of Foundational Machine-Learned Force Fields. *The Journal of Physical Chemistry C* **2025**, *129*, 9662–9669.
